# Nestin/CXCL12 immunohistochemistry and RNA sequencing map the bone marrow microenvironment in aplastic anemia

**DOI:** 10.1242/dmm.052564

**Published:** 2026-05-05

**Authors:** Astrid Beerlage, Carl P. Zinner, Jakob R. Passweg, Beatrice Drexler, Alexandar Tzankov

**Affiliations:** ^1^ Division of Hematology, University Hospital Basel, University of Basel, 4031 Basel, Switzerland; ^2^ Institute of Medical Genetics and Pathology, University Hospital Basel, University of Basel, 4031 Basel, Switzerland

**Keywords:** Aplastic anemia, Bone marrow niche, Bone marrow microenvironment, Nestin, CXCL12

## Abstract

Aplastic anemia (AA) is a rare bone marrow failure syndrome characterized by immune-mediated destruction of hematopoietic stem and progenitor cells (HSPCs). The contribution of the bone marrow microenvironment remains incompletely understood. Here, we analyzed 29 bone marrow biopsies from patients with moderate (mAA), severe (sAA) and very severe (vsAA) AA, along with 12 unaffected controls and seven subcortical pseudohypocellular samples. Immunohistochemistry for nestin and CXCL12 was performed to quantify stromal niches. RNA sequencing was carried out to investigate immune and niche-related gene expression patterns.

Patients with sAA exhibited a significantly increased number of nestin^+^ niches compared to patients with mAA and controls. CXCL12^+^ niches showed no significant differences between groups. RNA sequencing revealed upregulation of immune response genes, as well as pathways related to interferon-gamma signaling, JAK-STAT3 activation and antigen presentation. Downregulated genes and pathways pointed to impaired DNA repair, cell cycle regulation and epigenetic stability. Our findings support a model in which AA pathogenesis is driven by immune injury and compensatory, yet dysfunctional, stromal remodeling. These data underline the importance of the bone marrow microenvironment in AA.

## Teaser

**Summary:** In severe aplastic anemia, nestin-positive niches increase but CXCL12 niches remain stable. RNA sequencing reveals immune activation and disrupted cellular pathways, linking immune injury with microenvironmental remodeling.

## INTRODUCTION

Aplastic anemia (AA) is a rare and life-threatening disorder characterized by bone marrow failure, leading to pancytopenia. The incidence of AA varies geographically, with higher prevalence observed in Asia than in Europe and North America (Camitta et al., 1976; Montane et al., 2008). Although environmental toxins, radiation exposure, viral infections and autoimmune conditions have been implicated in disease onset, most cases remain idiopathic. Unlike inherited bone marrow failure syndromes, which typically present in childhood, AA is believed to be primarily immune mediated and can occur at any age (Adhikari and Mandal, 2019; Young and Maciejewski, 1997).

Patients with AA typically present with bicytopenia or pancytopenia accompanied by bone marrow hypocellularity. The immune-mediated destruction of hematopoietic stem and progenitor cells (HSPCs) is driven by autoreactive cytotoxic T cells, which secrete pro-inflammatory cytokines such as interferon-gamma (IFN-γ) and tumor necrosis factor-alpha (TNF-α; also known as TNF) (Wang and Liu, 2019). These cytokines contribute to HSPC apoptosis via the FAS/FAS ligand pathway and increased expression of major histocompatibility complex (MHC) class I molecules (Dubey et al., 2005; Zeng and Katsanis, 2015). Consequently, immunosuppressive therapy, including anti-thymocyte globulin (ATG) and calcineurin inhibitors, remains a cornerstone of treatment (Camitta et al., 1982).

The bone marrow niche is a specialized microenvironment that regulates HSPC maintenance, differentiation and protection from damage. Disruptions in the niche, including loss of osteoblasts and perivascular stromal cells, have been observed in patients with AA, suggesting that microenvironmental defects are contributory (Wang and Liu, 2019). Studies in murine models have demonstrated that osteoblast depletion leads to a reduction in HSPCs and impaired hematopoiesis (Visnjic et al., 2004). Similarly, human bone marrow samples from patients with AA show a decline in endosteal, vascular and perivascular stromal cells (Wu et al., 2015).

Mesenchymal stem/stromal cells (MSCs) are essential regulators of the bone marrow niche, in which they support hematopoiesis and contribute to the maintenance, migration and retention of HSPCs. In addition to their structural role, MSCs exert important immunomodulatory functions through interactions with immune cells, including T-helper cells and associated cytokines, thereby influencing the immune balance within the bone marrow microenvironment. This further implicates the bone marrow microenvironment in AA pathogenesis (Li et al., 2012). Nevertheless, the precise role of MSCs in AA remains debated, as some studies suggest that MSC function and phenotype remain largely preserved in this condition (Bueno et al., 2014).

One of the key mechanisms through which MSCs regulate HSPCs is the secretion of the chemokine CXCL12, also known as stromal cell-derived factor 1 (SDF-1). CXCL12 plays an essential role in maintaining a quiescent hematopoietic stem cell pool (Sugiyama et al., 2006). As a major source of CXCL12 in the bone marrow, MSCs, together with stromal cells, endothelial cells and osteoblasts, establish a chemotactic gradient that directs HSPC migration and promotes their retention within the niche. In AA, functional alterations in MSCs have been reported, including the downregulation of CXCL12 expression, which may contribute to impaired hematopoiesis (Chao et al., 2015).

Nestin, a type IV intermediate filament protein, is expressed in various stem and progenitor cells, including MSCs and perivascular stromal cells. Nestin-expressing stromal cells support HSPC maintenance and provide critical signals for niche function. Increased nestin expression has been observed in response to physiological stress, inflammation and hematopoietic regeneration. However, its role in disease states is complex. Loss of nestin^+^ MSCs has been implicated in myeloproliferative neoplasms (Arranz et al., 2014), while elevated nestin^+^ MSC activity has been linked to acute myeloid leukemia (AML) progression by enhancing antioxidant defense and chemotherapy resistance (Forte et al., 2020).

In this study, we analyzed nestin^+^ stem cell niches and CXCL12 expression in acquired AA. We correlated these findings with RNA-sequencing data to further elucidate the pathophysiological role of the bone marrow microenvironment in this condition.

## RESULTS

### Baseline characteristics

The study population covered all severity forms of acquired aplastic anemia [*n*=8 for moderate AA (mAA), *n*=11 for severe AA (sAA), *n*=5 for very severe AA (vsAA)]. Blood counts at first biopsy, and results of flow cytometry testing for paroxysmal nocturnal hemoglobinuria (PNH), cytogenetic abnormalities and next-generation sequencing (NGS) are shown in Table 1. None of the patients had a history suggestive of an inherited bone marrow failure, and all genetic variants present at a variant allelic frequency (VAF) of ∼50% (therefore suggestive of germline mutations) have not been described as pathological in ClinVar (genetic variants database from the National Institutes of Health) and were therefore interpreted as variants of unknown significance. During follow-up, two patients progressed to either a myelodysplastic syndrome (MDS; MDS-EB2) or AML (Table 2). At the time of transformation, both patients gained cytogenetic and molecular abnormalities.

**Table 1.** Patient characteristics at first biopsy

	(v)sAA (*n*=16)	mAA (*n*=8)
Blood count	Mean (minimum/maximum; *n*)	Mean (minimum/maximum; *n*)
Hemoglobin (g/l)	75 (54/92; 14)	92 (68/107; 7)
Reticulocytes (10^9^/l)	22 (4/57; 14)	51 (18/86; 6)
Thrombocytes (10^9^/l)	17 (2/53; 14)	51 (20/116; 7)
Leucocytes (10^9^/l)	1.9 (0.7/4.6; 14)	3.0 (2.0/4.6; 7)
Neutrophils (10^9^/l)	0.4 (0.0/1.7; 14)	1.0 (0.7/1.6; 6)
PNH clone	Positive (6, 43%); minor (1), major (5), no PNH clone (7), no data (3)	Positive (1, 13%); major (1), no PNH clone (5), no data (2)
Cytogenetic abnormalities	Normal (13), no data (3)	Normal (5), no data (3)
Molecular diagnostics	Any mutation: 3 (21%) Unremarkable (7), no data (4) Patient 11: *NF1* (VAF 51%, germline) Patient 16: *RUNX1* (VAF 50%, germline not excluded) Patient 24: *DNMT3A* (VAF 37%), *CBL* (two variants, VAF 7% and 49%)	Any mutation: 0 (0%) Unremarkable (3), no data (5)

PNH, paroxysmal nocturnal hemoglobinuria; VAF, variant allelic frequency; (v)sAA, (very) severe aplastic anemia.

**Table 2.** Patient characteristics at follow-up biopsies

	Patient 2	Patient 5		Patient 6	Patient 8
		Follow-up 1	Follow-up 2		
Initial diagnosis	mAA	mAA		sAA	sAA
Previous treatment	ATG+CNI	No previous treatment		CNI, ATG+CNI	ATG+CNI+TPO-RA
Time from initial diagnosis to follow-up biopsy	7 years	6 weeks	9 weeks	2 years, 11 months	6 months
Best response to previous treatment	Partial response	n.a. (progression from mAA to sAA)		No response	Partial response
Diagnosis	AML	vsAA	vsAA	MDS-EB2	sAA
Blood count					
Hemoglobin (g/l)	63	66	75	103	88
Reticulocytes (10^9^/l)	10	17	20	80	22
Thrombocytes (10^9^/l)	56	4	1	48	9
Leucocytes (10^9^/l)	1.6	1.1	1.0	3.1	1.6
Neutrophiles (10^9^/l)	0.2	0.2	0.2	0.5	0.7
Cytogenetic abnormalities	−7, del(3p24.4)	none	n.a.	45,XY,−7; 46,XY,−7,+21	n.a.
Molecular diagnostics	*ASXL1*, *GATA2*, *SRSF2*	n.a.	n.a.	*PTPN11*, *RUNX1*	n.a.

AML, acute myeloid leukemia; ATG, horse anti-thymocyte globulin; CNI, calcineurin inhibitor; mAA moderate aplastic anemia; MDS, myelodysplastic syndrome; n.a., not applicable; sAA, severe aplastic anemia; TPO-RA, thrombopoietin receptor agonist; vsAA, very severe aplastic anemia.

Infectious diseases [hepatitis A, B and C (HAV, HBV and HCV, respectively); human immunodeficiency virus (HIV); parvovirus B19] were excluded at the time of diagnosis. One patient showed seronegative, autoimmune hepatitis before the diagnosis of pancytopenia and was classified as having hepatitis-associated AA.

Patients received standard immunosuppressive therapies with calcineurin inhibitors combined with ATG and thrombopoietin receptor agonist (TPO-RA) or allogeneic hematopoieitic cell transplantation (summarized in Table S1); one patient was monitored without treatment. Survival data for severe and non-severe AA are shown in Fig. S1.

### Patients with sAA have more nestin^+^ niches than do patients with mAA and unaffected controls

**Fig. 1.**
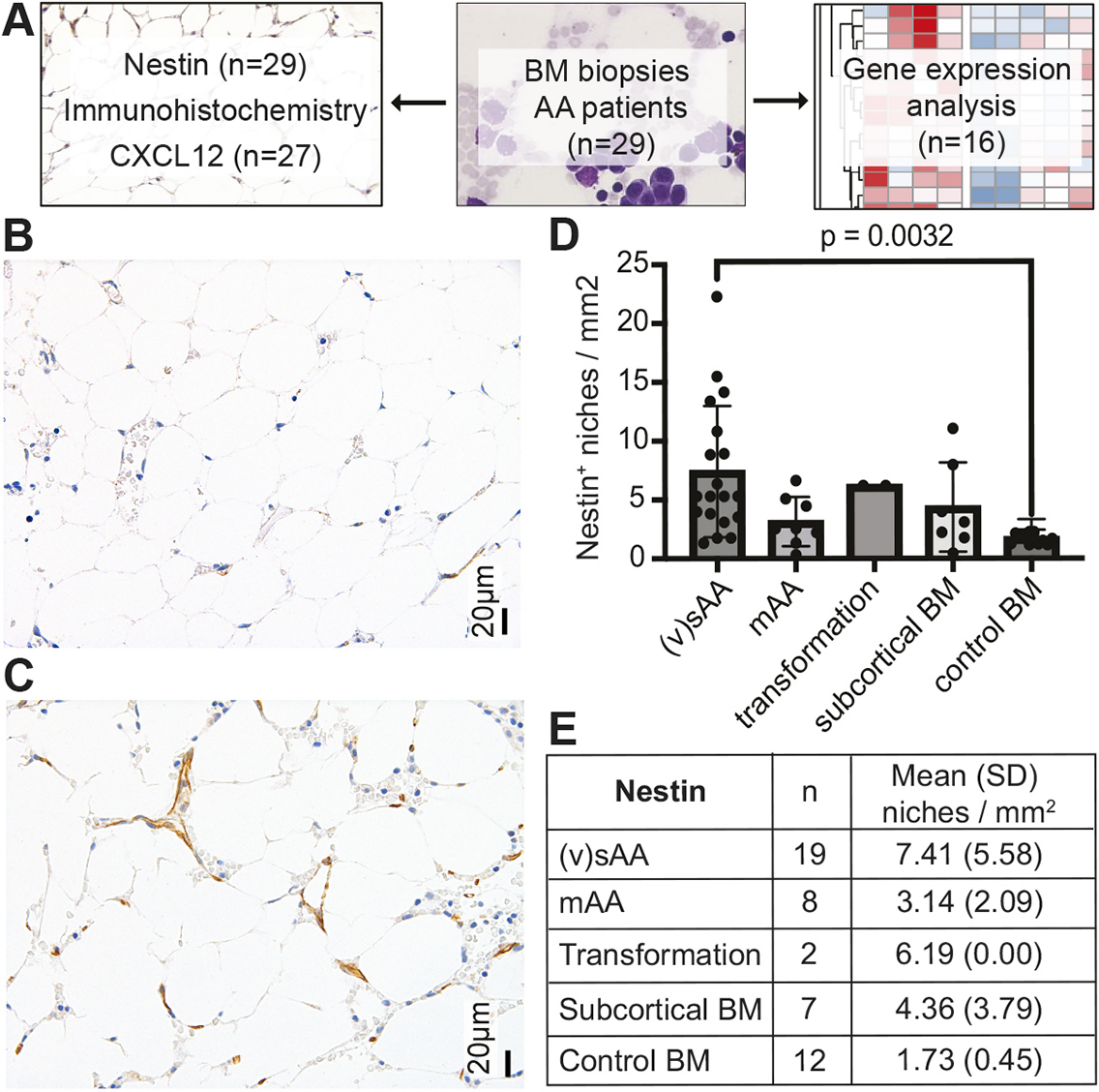
**Study design and immunohistochemistry for nestin.** (A) Patient samples were analyzed by immunohistochemistry for nestin and CXCL12 and by RNA analysis for gene expression. AA, aplastic anemia; BM, bone marrow. (B,C) Example of low nestin expression in a control BM sample (B) and high nestin expression in a patient with severe AA (sAA) (C). (D,E) Bar plot showing mean with s.d. and results of Tukey's multiple comparison test (D) and overview table (E) of quantification of nestin staining in 48 analyzed samples. mAA, moderate AA; (v)sAA, (very) severe AA.

Patients with mAA showed similar nestin^+^ niches to unaffected controls and subcortical biopsies (Fig. 1). Conversely, the number of nestin^+^ niches was higher in patients with sAA than in controls (*P*=0.0032). Patients whose AA transformed to myeloid malignancies also showed a (non-statistically significant) higher amount of nestin^+^ niches. No clinically abnormal features were noted for the outlier with 22.3 nestin^+^ niches/mm^2^. Representative immunohistochemistry results are shown in Fig. 1.

### CXCL12^+^ niches are similar in patients with sAA and mAA and controls

**Fig. 2.**
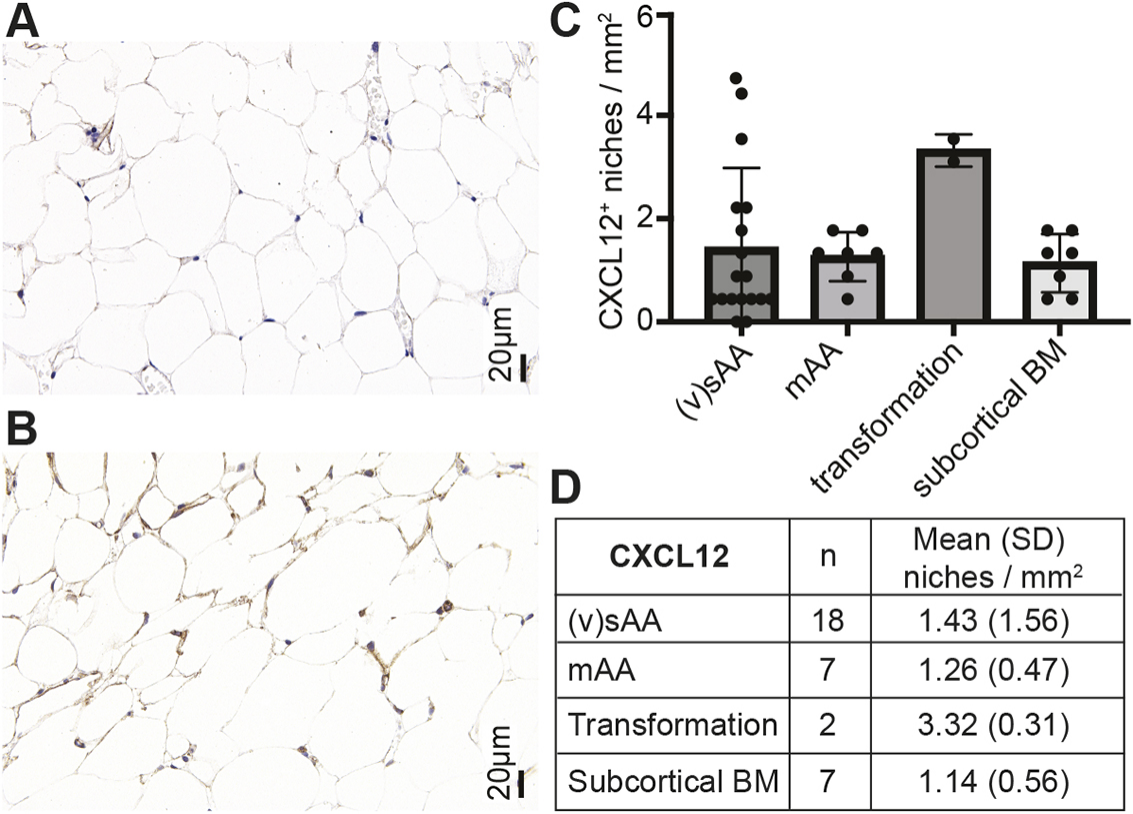
**Immunohistochemistry results for CXCL12.** (A,B) Examples of low CXCL12 expression in a control BM sample (A) and high CXCL12 expression in a patient with sAA (B). (C,D) Bar plot showing mean with s.d. (C) and overview table (D) of quantification of CXCL12 staining in 35 analyzed samples.

Immunohistochemistry did not show significant differences in the presence of CXCL12^+^ niches between the different subgroups (Fig. 2). However, there was a trend towards a higher niche density in cases with malignant transformation. For example, patient 2 showed increased CXCL12^+^ niches from 1.7/mm^2^ to 3.1/mm^2^. Although CXCL12^+^ niches were similar in the mAA, transformed AA and cortical bone marrow groups, the sAA cohort showed three outliers with markedly increased CXCL12. One of these patients had an unremarkable clinical history. The second patient had a history of autoimmune hepatitis with associated sAA and showed a *DNMT3A* mutation and *CBL* variant. This patient did not show a concurrent increase in nestin^+^ niches (1.33 niches/mm^2^). The third case was a follow-up biopsy from a patient who showed no response to initial treatment; the CXCL12^+^ niches/mm^2^ increased from 1.8 niches/mm^2^ to 5.3 niches/mm^2^. Representative immunohistochemistry results are shown in Fig. 2. We examined the relationship between the gene expression and immunohistochemistry of CXCL12 by correlating the binary logarithm of the normalized counts with (CXCL12^+^ niches/mm^2^) in the differential expression framework. This Pearson-type analysis yielded a 2.98-fold increase in the gene expression per unit increase in (CXCL12^+^ niches/mm^2^), with statistical significance of *P*=0.0028.

### Immune activation and inflammatory signaling in AA

**Fig. 3.**
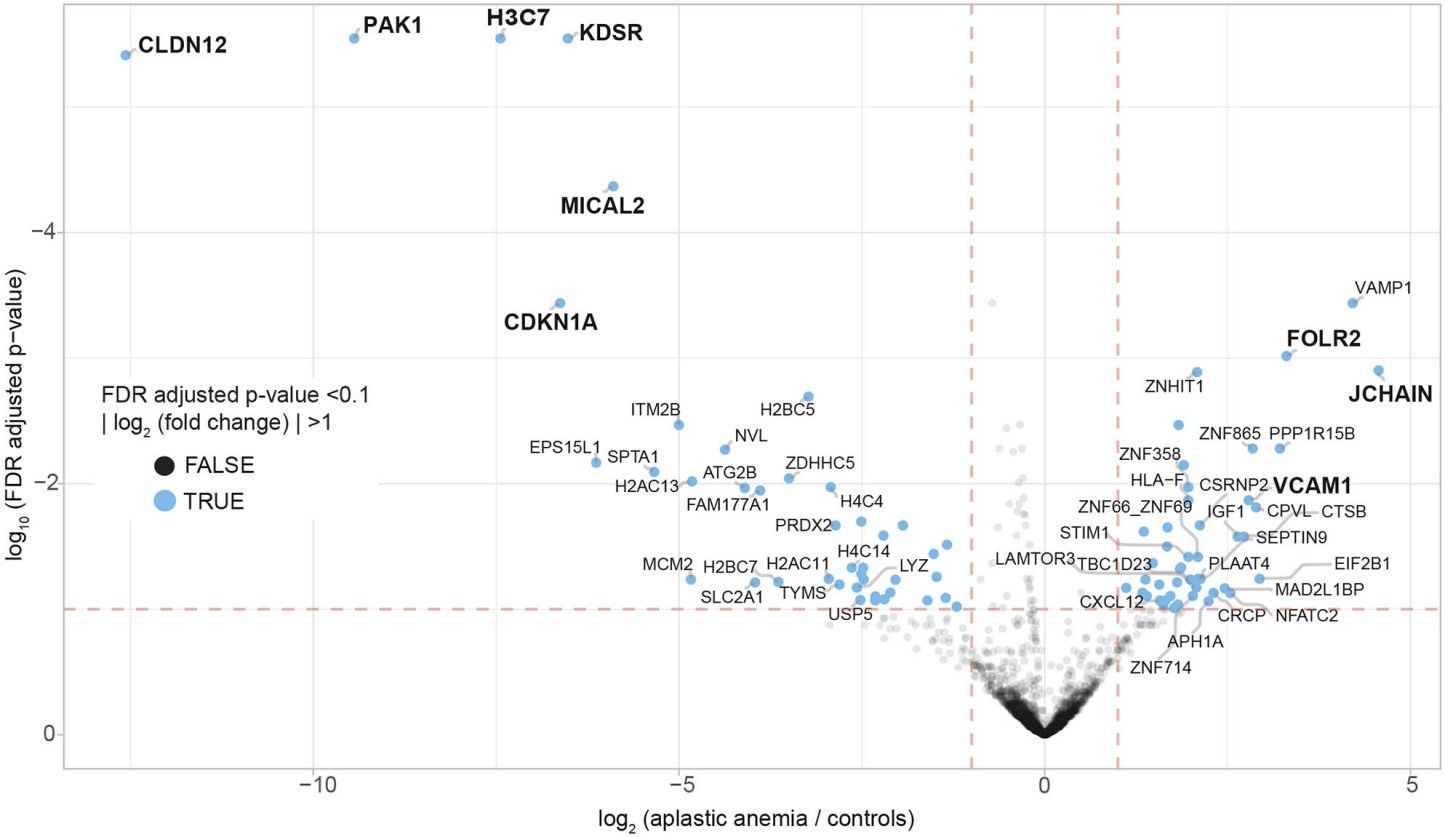
**Gene expression analysis.** Volcano plot of the top upregulated and downregulated genes in patients with AA in comparison to unaffected controls. Top upregulated genes include the immune-related genes *JCHAIN*, *FOLR2* and *VCAM1*. Top downregulated genes include genes involved in cell cycle regulation (*PAK1*, *CDKN1A*). FDR, false discovery rate.

RNA-sequencing data (GSE319656) were visualized using a volcano plot (Fig. 3) and heatmap (Fig. S2), demonstrating distinct gene expression profiles between controls and patients with mAA and sAA. Principal component analysis showed the distinct patient groups (Fig. S3).

AA upregulated key immune-related genes, including *JCHAIN*, *FOLR2* and *VCAM1* (Fig. 3). *JCHAIN* encodes the joining chain of immunoglobulins, which plays a role in mucosal immunity and B-cell activation. Upregulation of *FOLR2*, a marker of M2 macrophages, suggests a shift toward a pro-inflammatory or immunomodulatory response. *VCAM1*, encoding a critical cell adhesion molecule, was also upregulated, aligning with Kyoto Encyclopedia of Genes and Genomes (KEGG) pathway enrichment of cell adhesion molecules and graft-versus-host disease (GvHD) pathways, indicating increased immune cell trafficking and hematopoietic niche alterations in AA.

**Fig. 4.**
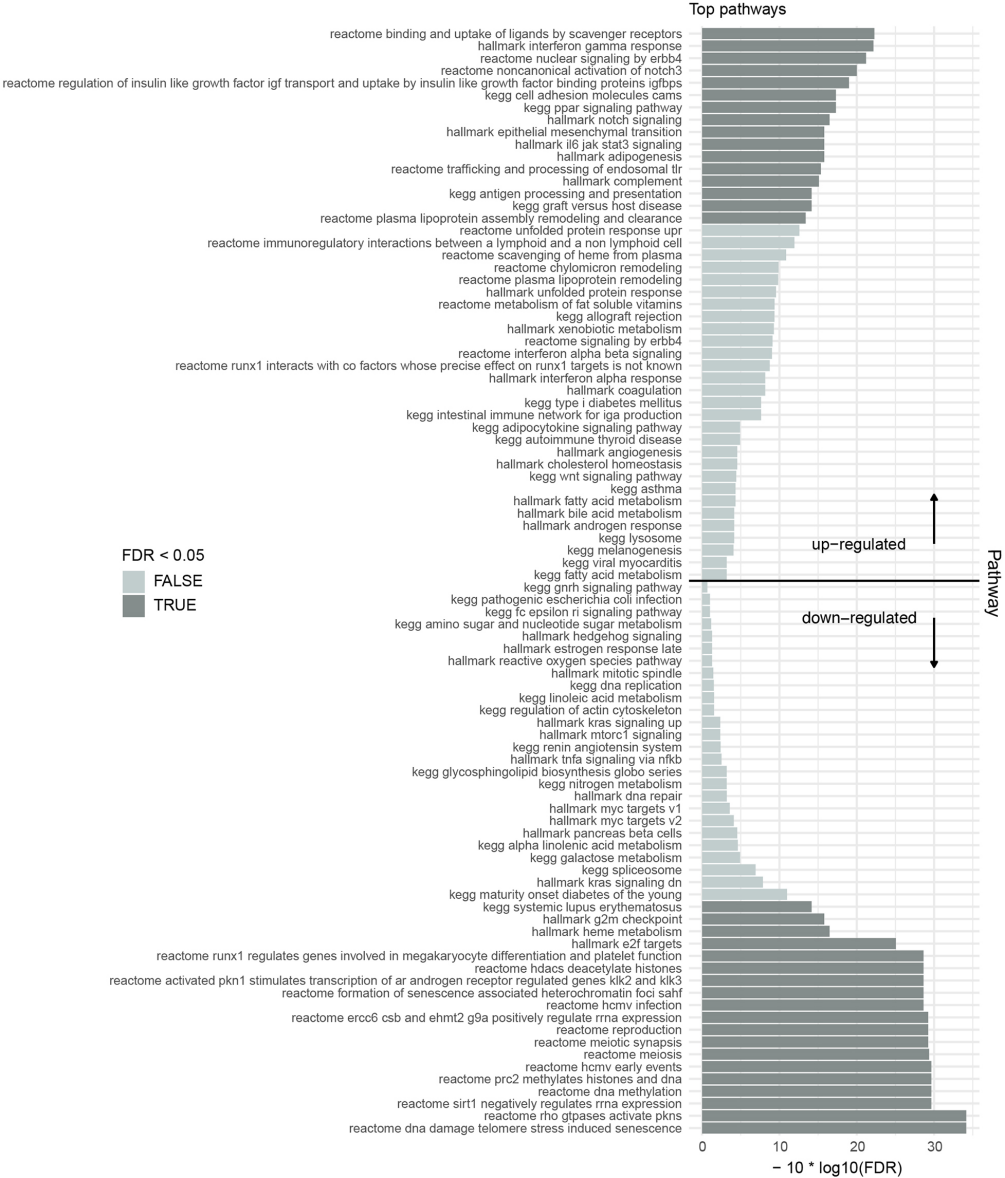
**Gene set collection analysis.** Combined gene set collection analysis [Hallmark, Reactome, Kyoto Encyclopedia of Genes and Genomes (KEGG)] showing the top upregulated and downregulated pathways of all AA versus control samples. Upregulated pathways underline the inflammatory bone marrow microenvironment as well as a dysregulated immune environment. Downregulated pathways align with impaired DNA repair, cell cycle regulation and epigenetic stability in AA.

In parallel, Hallmark pathway analysis (Fig. 4) showed significant enrichment of IFN-γ response, JAK-STAT3 signaling and Notch signaling, all contributing to immune activation and bone marrow inflammation. The upregulation of antigen processing and presentation pathways further supports a heightened immune response against HSPCs.

### Downregulation of DNA repair, cell cycle regulation and hematopoiesis

Significant downregulation of genes associated with DNA repair, hematopoietic regulation and cell cycle progression was observed. *CLDN12*, encoding a tight junction protein, was downregulated. *PAK1*, involved in cytoskeletal remodeling and cell cycle progression, was significantly suppressed. *H3C7*, encoding a core histone protein, and *KDSR*, encoding an enzyme involved in sphingolipid metabolism, were downregulated. *MICAL2*, encoding an oxidoreductase implicated in actin cytoskeleton remodeling, was suppressed. *CDKN1A*, a key cell cycle inhibitor, was downregulated (Fig. 3).

Pathway analysis showed suppression of DNA methylation, PRC2 histone modification and HDAC activity (Reactome pathway analysis). Additionally, it showed downregulation of telomere stress-induced senescence pathways (Fig. 4).

### Metabolic and microenvironmental shifts in AA

KEGG and Hallmark pathway analysis revealed enrichment of adipogenesis and epithelial-mesenchymal transition (EMT) pathways (Fig. 4). The upregulation of PPAR signaling and plasma lipoprotein metabolism aligns with these findings.

## DISCUSSION

Our findings provide further insight into the role of the bone marrow microenvironment in acquired AA. The immune-mediated destruction of HSPCs is well established, with autoreactive T cells and pro-inflammatory cytokines such as IFN-γ and TNF-α driving bone marrow failure (Young, 2018; Wu et al., 2025). However, increasing evidence suggests that the bone marrow niche is not merely a passive bystander but actively contributes to disease progression.

Our immunohistochemical analysis revealed a significant increase in nestin^+^ niches in patients with sAA and in patients who later progressed to having myeloid malignancies. Nestin-expressing MSCs are known to support HSPC maintenance and quiescence (Mendez-Ferrer et al., 2010) but have also been shown to enhance leukemia cell survival in AML (Forte et al., 2020). In a mouse model of rheumatoid arthritis, a significant TGF-β-mediated expansion of nestin^+^ cells could be shown early after disease induction, and deletion of the TGF-β receptor attenuated joint destruction. Increased nestin^+^ niches in sAA may, therefore, indicate an attempt of the bone marrow microenvironment to improve HSPC support as well as part of a more general auto-immune inflammatory response in the bone marrow. This contrasts with the decreased number of nestin^+^ niches in the setting of alloreactive cytopenias that can be seen in the context of acute GvHD (Medinger et al., 2015), the mechanisms of which seem not to affect HSPCs.

CXCL12 production by MSC progenitors is required for HPSC maintenance (Greenbaum et al., 2013) and also the retention of regulatory T cells (Zou et al., 2004). Elevated CXCL12 levels and quantitative changes in CXCL12^+^ cells have been described in the context of MDS and AML with myelodysplasia-related changes (Abe-Suzuki et al., 2014; Prummel et al., 2025), consistent with the findings of the two included patients with transformation in our study. The absence of significant changes in CXCL12 expression and distribution across AA subtypes, except in cases of malignant transformation, suggests that although the niche is altered, key HSPC retention signals remain largely intact. This reinforces that immune destruction and cell-intrinsic defects, rather than niche dysfunction alone, drive disease progression.

RNA-sequencing analysis further supports the involvement of immune dysregulation and microenvironmental alterations in AA. Upregulation of *JCHAIN*, *FOLR2* and *VCAM1* highlights an ongoing inflammatory response and changes in cell adhesion properties within the bone marrow. *JCHAIN* expression suggests enhanced immune activity, while expression of *FOLR2*, a marker of M2 macrophages, may indicate a pro-inflammatory response perpetuating immune damage (Chen et al., 2023). The upregulation of *VCAM1*, a key mediator of HSPC-stromal interactions, aligns with KEGG pathway enrichment of cell adhesion molecules (Li et al., 2018) and GvHD pathways, further emphasizing the dysregulated immune environment. The observed *in situ* changes with respect to numbers of nestin^+^ niches were not reflected by gene expression profiling (GEP). This may be explained by the overall low numbers of niches and the known lack of linear dependency of mRNA and protein levels and half-lives. Regarding CXCL12, both immunhistochemistry and GEP analysis did not show significant differences between unaffected individuals and patients with AA. Although an increase in *CXCL12* was perceptible upon supervised GEP analysis, *CXCL12* was not among the 20 top upregulated genes, and its upregulation lacked statistical significance (data, therefore, not shown in detail).

The function of *CLDN12*, encoding a tight junction protein, in the bone marrow is not well described. However, CLDN12 deficiency has been shown to impair transendothelial migration of myeloid-derived suppressor cells (Cao et al., 2022). Therefore, its suppression in AA may impact bone marrow endothelial integrity and stromal interactions. Suppression of *MICAL2* suggests impaired cellular migration and adhesion in the bone marrow niche. Both factors potentially contribute to the disrupted bone marrow microenvironment that was observed.

*PAK1* downregulation aligns with the observed downregulation of G_2_M checkpoint and chromosome condensation pathways. Downregulation of *H3C7* proposes compromised niche integrity and stromal interaction and disrupted chromatin regulation. Similarly, *PAK1* downregulation and suppressed G_2_M checkpoint pathways imply impaired cytoskeletal dynamics and cell cycle progression, potentially contributing to HSPC dysfunction. The suppression of DNA repair and epigenetic regulation pathways, including histone modifications, further supports the hypothesis that epigenetic dysregulation contributes to HSPC exhaustion (Laugesen and Helin, 2014). Downregulation of the cell cycle inhibitor *CDKN1A* (p21) first seems paradoxical in this context. Taken together, these results suggest that cell cycle arrest may occur through alternative mechanisms, such as apoptosis induction rather than senescence-associated p21 activation.

Metabolic shifts observed in our pathway analysis, including upregulation of PPAR signaling and adipogenesis, reflect noticeable changes in bone marrow stromal composition in AA. These findings align with reports of increased marrow adiposity in AA and its potential role in exacerbating hematopoietic failure (Tripathy et al., 2014). Enriching EMT pathways further suggests that mesenchymal cells within the niche may undergo adaptive changes in response to stress (Kalluri and Weinberg, 2009).

Our results support a model in which immune-mediated HSPC destruction and concurrent alterations in the bone marrow niche drive AA pathogenesis. The observed increase in nestin^+^ niches suggests an attempt at compensation that ultimately fails in severe disease. Meanwhile, CXCL12 expression remains largely unaltered except in cases of malignant progression, in which it may reflect niche remodeling in response to clonal evolution.

Our study is limited by the small sample size and retrospective data collection, which might have impaired the data quality of the gene expression analysis through RNA sequencing. Nevertheless, our findings underline the importance of integrating immune and stromal interactions in the pathophysiology of AA. By elucidating the role of nestin^+^ MSCs and key molecular pathways in disease progression, we provide a foundation for future research to investigate the functional consequences of these niche alterations and develop targeted therapies to restore bone marrow homeostasis in AA.

## MATERIALS AND METHODS

### Study population

We collected 29 formalin-fixed, EDTA-decalcified and paraffin-embedded bone marrow biopsies from patients diagnosed with AA at the Institute of Pathology at the University Hospital Basel, Basel, Switzerland, between 2013 and 2020. Most samples (*n*=24) were taken during the initial work-up, and five biopsies were taken during follow-up, showing persisting AA in three cases and a transformation into a myeloid malignancy in two cases (AML and MDS-EB2). Twelve bone marrow samples from healthy donors served as controls. As a second control group, we analyzed seven subcortical biopsies with physiological pseudoaplastic bone marrow to account for the generally very small cell numbers in AA. Patients had regular follow-up visits at the Department of Hematology at the University Hospital Basel, and clinical data were collected by chart review until May 2023. All patients included in this study provided written consent for the use of clinical data and biomaterial prior to treatment initiation. The local ethics committee (Ethikkomission Nordwest- und Zentralschweiz) approved the study (EKNZ Project-ID 2018-00281), and all clinical investigations were conducted according to the principles expressed in the Declaration of Helsinki.

### Diagnosis

The Camitta Criteria (Camitta et al., 1982) defined diagnosis and severity. For sAA, the following had to be fulfilled: bone marrow cellularity of <30% and two of three of the subsequent criteria: reticulocytes <20×10^9^/l (manual) or <10^9^/l (automated), absolute neutrophil count (ANC) <0.5×10^9^/l and platelets <20×10^9^/l. If patients fulfilled these criteria and additionally had an ANC <0.25×10^9^/l, they were classified as having vsAA. mAA was diagnosed in cases with hypocellular marrow and cytopenias in at least two of three peripheral blood cell lines (ANC <1.2×10^9^/l and ≥0.5×10^9^/l, platelet count <70×10^9^/l, absolute reticulocyte count <60×10^9^/l). Inherited bone marrow failure syndromes and secondary causes of cytopenia, including malignancy (particularly MDS), viral infections and drug exposure, were excluded based on detailed patient and family histories and comprehensive serological testing (HAV, HBV, HCV, cytomegalovirus, Epstein–Barr virus, HIV and parvovirus B19). As none of the patients had clinical features suggestive of inherited bone marrow failure, targeted germline genetic testing was not performed. Nevertheless, the applied NGS panel included several genes commonly assessed in hereditary bone marrow failure panels, including *RUNX1*, *GATA2*, *ETV6* and *CEBPA*.

### Flow cytometry as screening for a PNH clone

As part of the routine initial workup, peripheral blood was screened for the presence of a PNH clone. The presence of CD24^−^ and FLAER^−^ cells (granulocytes) or CD14^−^ and FLAER^−^ cells (monocytes) defined the presence of a PNH clone. A fraction of the parent population of >1% was defined as a major PNH clone, and a fraction of 0.1-1% was defined as a minor PNH clone. Additionally, erythrocytic PNH clones were identified by CD59 staining.

### Screening for genetic abnormalities by NGS and cytogenetic analysis

Some patients (especially those diagnosed after 2018) received cytogenetic and molecular analysis to rule out differential diagnosis of possible hypocellular MDS and germline predisposition. Metaphases were stained by G-banding and subsequently analyzed for numeric and structural abnormalities (conventional cytogenetics). NGS was performed for characteristic MDS-associated mutations with a 39-gene panel including *ABL1*, *ASXL1*, *BCR*, *BRAF*, *CALR*, *CBL*, *CEBPA*, *CHEK2*, *CSF3R*, *DNMT3A*, *EGLN1*, *EPOR*, *ETNK1*, *ETV6*, *EZH2*, *FLT3*, *GATA2*, *IDH1*, *IDH2*, *JAK2*, *KIT*, *KRAS*, *MPL*, *NF1*, *NPM1*, *NRAS*, *PDGFRA*, *PDGFRB*, *PTPN11*, *RUNX1*, *SETBP1*, *SF3B1*, *SH2B3*, *SRSF2*, *TET2*, *TP53*, *U2AF1*, *VHL* and *ZRSR2*. One patient's molecular testing was performed at an external center with a 36-gene panel vastly overlapping the first panel (*ASXL1*, *BCOR*, *BRAF*, *CALR*, *CBL*, *CEBPA*, *CSF3R*, *DNMT3A*, *ETV6*, *EZH2*, *FLT3*, *FLT3-ITD*, *GATA2*, *HRAS*, *IDH1*, *IDH2*, *JAK2*, *KIT*, *KRAS*, *MPL*, *NPM1*, *NRAS*, *PHF6*, *PRPF8*, *PTPN11*, *RUNX1*, *SETPB1*, *SF3B1*, *SH2B3*, *SRSF2*, *STAG2*, *TET2*, *TP53*, *U2AF1*, *WT1* and *ZRSR2*).

### Immunohistochemistry

Nestin staining was performed on all 48 study and control biopsies, applying the monoclonal antibody clone 10C2 from AbD Serotec (OBT1610) at a dilution of 1:50 for 12 min using an automated immunostainer (Benchmark, Ventana/Roche). For CXCL12 staining, the monoclonal antibody clone 79018 from R&D Systems (MAB350-100) was applied at a dilution of 1:400 with manual overnight incubation at 4°C. Owing to a lack of substance in deeper sections of the tissue blocks in one patient with sAA, one patient with mAA and 12 unaffected controls, CXCL12 staining could only be performed on 34 samples (27 patient biopsies, seven controls from subcortical bone marrow spaces). Antigen retrieval was achieved by cell conditioning (CC1 from Ventana/Roche) treatment for 40 min in the automated stainer for nestin, or microwave oven treatment for 30 min at 98°C with a pH 8 TEC buffer (TRIS/EDTA/citrate from BioLogo) for CXCL12. Visualization was achieved using the Ultraview detection system from Ventana/Roche for nestin and biotinylated horse anti-mouse IgG antibody BA-2000-1.5 from Vector Laboratories for CXCL12, with diaminobenzidine as a chromogen.

Scoring was performed by optical counting and considering the number of nestin^+^ or CXCL12^+^ niches (single cells or clusters of up to three cells) in the bone marrow samples. On average, 7.2 mm^2^ was evaluated, and results were normalized to 1 mm^2^.

### Gene expression analysis

GEP was performed on 25 samples with leftover tissue (20 AA and five controls) by HTG Molecular Diagnostics (Tucson, AZ, USA) according to established protocols. Lysates from samples were run on the HTG EdgeSeq Processor using the HTG EdgeSeq Immune Response Panel with an excess of nuclease protection probes (NPPs) complementary to their target. S1 nuclease removed unhybridized probes and RNAs, leaving NPPs hybridized to their targets in a one-to-one ratio. Samples were individually barcoded using a 16-cycle PCR reaction to add adapters and molecular barcodes, individually purified using AMPure XP beads (Beckman Coulter), and quantified using a KAPA Library Quantification kit (KAPA Biosystems). Libraries were sequenced on the Illumina SEQUENCER platform for quantification.

#### Quality control

Post-sequencing quality control targeted four sample metrics: insufficient RNA (QC0), insufficient read depth (QC1), insufficient expression variability (QC2) and incomplete digestion of gDNA by DNase (QC3). The sample failure mode QC2, defined as the median log2 (counts per million) of the negative control probes, clearly identified multiple samples as low quality. These samples were subsequently removed, and 16 samples were included in the final analysis [three mAA, 13 (v)sAA]. On a gene level, lowly expressed genes were filtered with the function filterByExpr of the R package edgeR (Chen et al., 2016).

#### Differential gene expression

Evaluation of the gene expression pipeline – including normalization, dispersion estimation and statistical testing – was carried out with the package DESeq2 (Love et al., 2014). When comparing samples with AA and controls, 23 genes were upregulated in AA with a false discovery rate (FDR)<0.05, and 43 genes were downregulated with an FDR<0.05, out of 3169 genes with nonzero total read count. LogFoldchanges were shrunk with the apeglm package (Zhu et al., 2019) for the volcano plot visualization. FDR adjustments were used with the Benjamini–Hochberg method (Benjamini and Hochberg, 1995).

#### Variance-stabilizing transformation

Normalized gene counts were further modified with variance-stabilizing transformation in the DESeq2 package. The transformed counts were utilized in principal component analysis, heatmap and box-plot visualizations. Principal components were calculated with the prcomp R function (part of the R stats package) using scaling and centering of inputs.

For the heatmap of gene counts, only those genes with an FDR<0.05 and logFoldchange>0.58 were selected. The gene counts were transformed to *z*-scores and clipped at three standard deviations. Hierarchical clustering of genes used Pearson correlation as a distance metric and complete linkage as method.

Box plots show the top 20 upregulated and downregulated genes for AA with FDR<0.05 and |logFoldchange|>1.

#### Gene set analysis

Gene set collections Hallmark (Liberzon et al., 2015), Reactome and KEGG (Subramanian et al., 2005) were retrieved with the msigdbr package in R (2022). Using the camera algorithm with quality weights (Ritchie et al., 2006, 2015), a competitive ranking of the gene sets was produced for the top 20 gene sets by FDR in each collection.

#### Cell type estimation

The abundance of cell types in the bulk RNA mixture was approximated with the CibersortX tool (Newman et al., 2019) using counts per million, absolute mode and batch mode. The abundances were tested for statistical differences using a quasi-binomial general linear model.

### Statistical analysis

Statistical analysis was done with SPSS (Version 28.0.1.0), Prism 9 (Version 9.5.1) and R (Version 4.2.3). Immunohistochemical staining was compared using ordinary one-way ANOVA, followed by Tukey's multiple comparison test for pairwise comparisons when appropriate.

### Artificial intelligence (AI) tools

ChatGPT (OpenAI) was used to assist with the preparation of an initial draft of the manuscript and to improve clarity, flow and readability of the text, including language refinement and shortening of selected passages. All AI-assisted content was manually reviewed, revised and approved by the authors. No generative AI tools were used for data collection, data analysis, interpretation of results, or image generation or modification. The authors take full responsibility for the content of the manuscript.

## Supplementary Material

10.1242/dmm.052564_sup1 Supplementary information
