# Supplementary material for: Nestin/CXCL12 immunohistochemistry and RNA sequencing map the bone marrow microenvironment in aplastic anemia
Source: Dis Model Mech. 2026 May 5;19(4):dmm052564. doi: 10.1242/dmm.052564 (PMC13225205; doi:10.1242/dmm.052564)
Supplement: Supplementary information [file dmm-19-052564-s1.pdf]

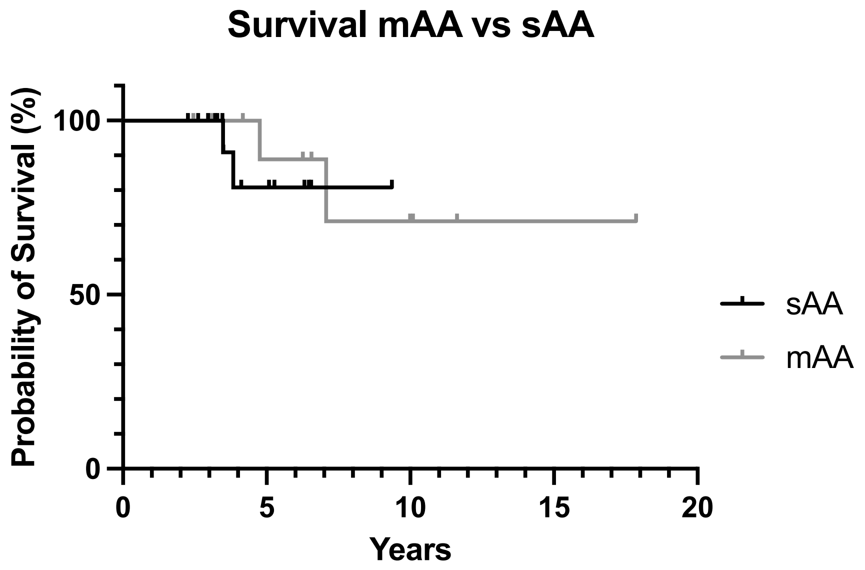

Fig. S1. Probability of survival in patients with moderate versus (v)sAA

Disease Models & Mechanisms • Supplementary information

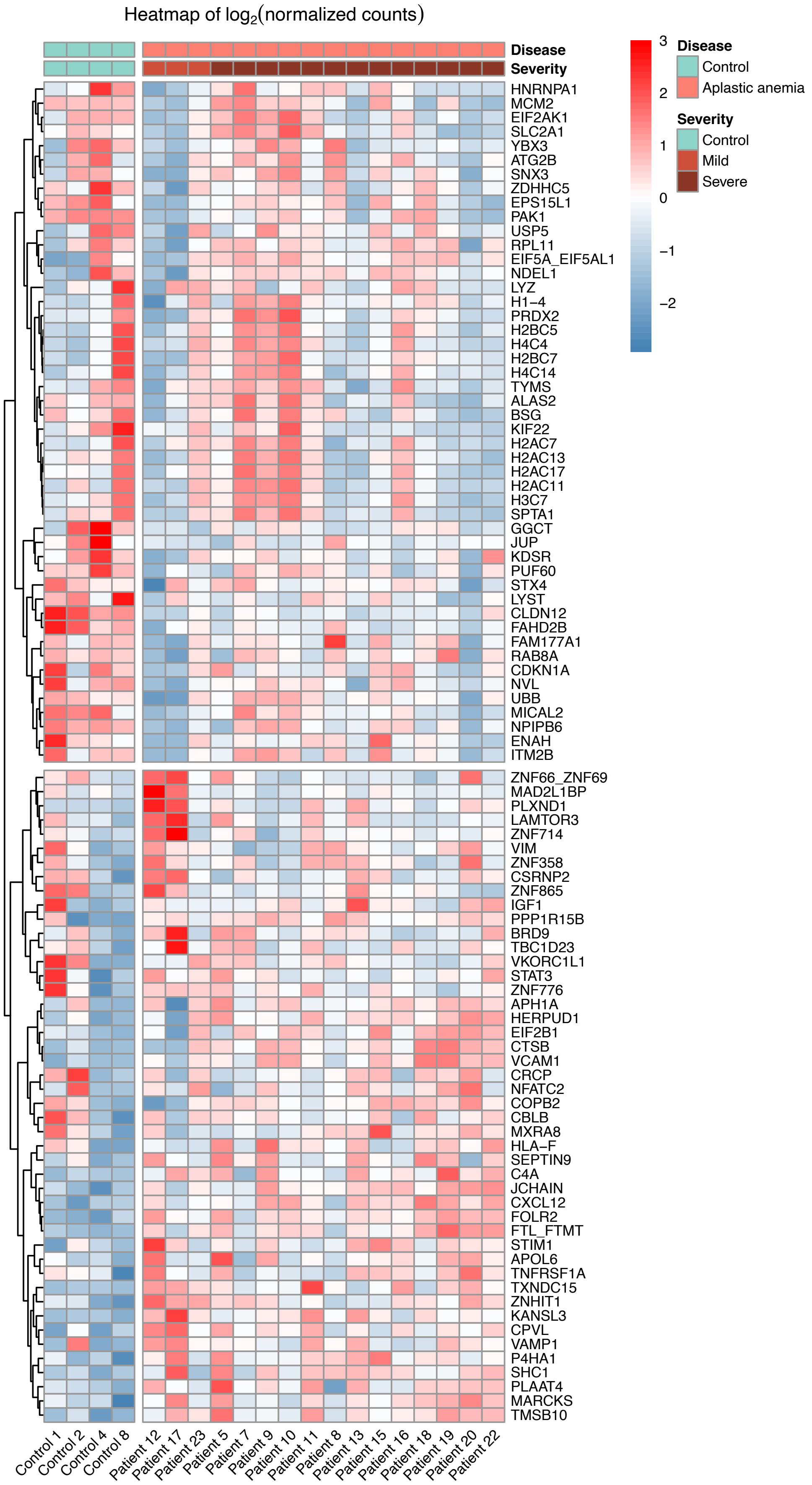

Fig. S2. Clustered heatmap of top up- and downregulated gene

Disease Models & Mechanisms • Supplementary information

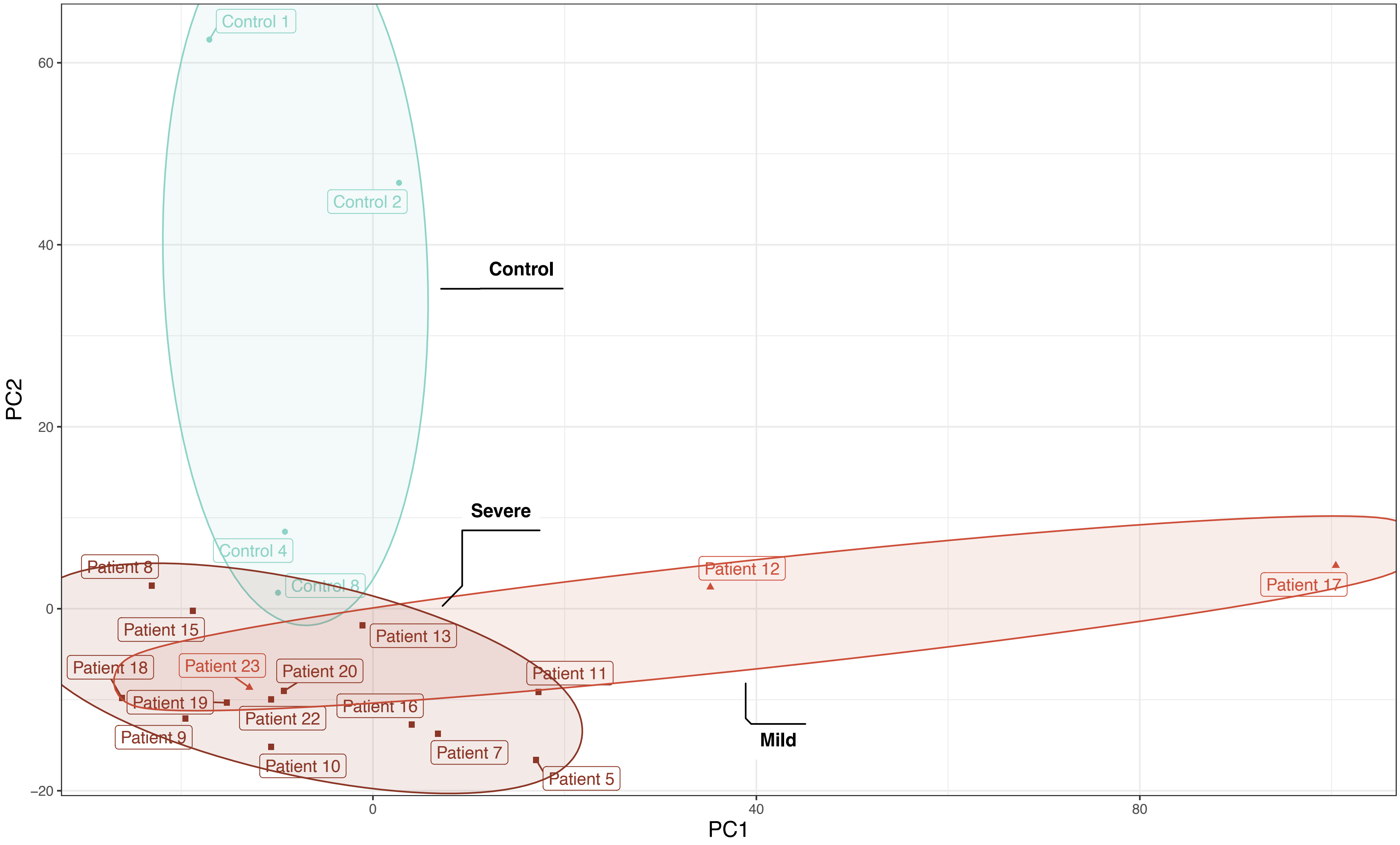

Fig. S3. Clustered heatmap of top up- and downregulated gene

Disease Models & Mechanisms • Supplementary information

Table S1. Treatment and best response to treatment for mAA and (v)sAA patients.

	SAA/ vSAA (n=15, no data available for 1 patient)	MAA (n=8)
**Best response**	CR (11, 73%)<br>PR (4, 27%)	CR (5, 63%)<br>PR (2, 25%)<br>No response (1, 13%)
**Treatment**		
First line therapy	CNI (1, 7%)<br>CNI + TPO-RA (1, 7%)<br>ATG + CNI (6, 40%)<br>ATG + CNI + TPO-RA (5, 33%)<br>Allogeneic HCT (2, 13%)	CNI (3, 36%)<br>CNI + TPO-RA (1, 13%)<br>ATG + CNI (2, 25%)<br>ATG + CNI + TPO-RA-agonist (1, 13%)<br>Allogeneic HCT (1, 13%)<br>No therapy (1, 13%)
2nd line therapies	CNI + TPO-RA (2, 40%)<br>ATG + CNI (1, 20%)<br>Allogeneic HCT (2, 40%)	TPO-RA (1, 20%)<br>CNI + TPO-RA (3, 60%)<br>Chemotherapy (after transformation; 1, 20%)
Later therapies		ATG + CNI (1, 33%)<br>ATG + CNI + TPO-RA (1, 33%)<br>Allogeneic HCT (1, 33%)

CR: complete remission, PR: partial remission, CNI: calcineurin inhibitor, TPO-RA: thrombopoietin receptor agonist, ATG: horse anti-thymocyte globulin, HCT: hematopoietic cell transplantation

Disease Models & Mechanisms • Supplementary information
